# Coeliac disease and risk for other autoimmune diseases in patients with Williams-Beuren syndrome

**DOI:** 10.1186/1471-2350-15-61

**Published:** 2014-05-23

**Authors:** Stefano Stagi, Elisabetta Lapi, Maria Gabriella D’Avanzo, Giancarlo Perferi, Silvia Romano, Sabrina Giglio, Silvia Ricci, Chiara Azzari, Francesco Chiarelli, Salvatore Seminara, Maurizio de Martino

**Affiliations:** 1 Department of Health Sciences, University of Florence, Anna Meyer Children’s University Hospital, Florence, Italy; 2 Genetics and Molecular Medicine Unit, Anna Meyer Children’s University Hospital, Florence, Italy; 3 Medical Genetics Unit, San Giuseppe Moscati Hospital, Avellino, Italy; 4 Department of Paediatrics, University of Chieti, Chieti, Italy

## Abstract

**Background:**

A higher prevalence of coeliac disease (CD) has been reported in patients with Williams-Beuren syndrome (WBS), though coexistence with other autoimmune diseases has not been evaluated.

Objective**:** The aim of this study was to examine the prevalence of the more frequent autoimmune diseases and organ- and non-organ specific autoantibodies in WBS.

**Methods:**

We longitudinally analysed 46 WBS patients to evaluate the prevalence and co-occurrence of the major autoantibodies and HLA typing for CD diagnosis. These data were compared with healthy age- and sex-matched controls and Down (DS) and Turner (TS) syndrome patients.

**Results:**

CD was diagnosed in one (2.2%) WBS patient; this differed significantly from DS and TS (respectively, 10.5% and 9.4%; P < 0.005) but not from healthy controls (0.6%; P = NS). However, no patients with WBS showed anti-thyroid antibodies or other organ- and non-organ specific autoantibodies, which differed significantly from DS (respectively, 10.5% and 7.0%; P < 0.005) and TS (respectively, 9.4% and 9.3%; P < 0.005) patients but not from healthy controls (1.1% and 2.3%). The frequencies of CD-specific HLA-DQ heterodimers were not significantly higher than controls, even though the WBS patients more frequently carried the DQA1*0505 allele (57% *vs.* 39%; P < 0.05).

**Conclusions:**

CD may not be more frequent in patients with WBS. In fact, no evidence of a significantly higher prevalence of other autoimmune diseases or positivity of the main organ and non-organ specific autoantibodies was found in WBS, such as showed in the healthy controls and unlike by the patients with Turner or Down syndrome. This should prompt us to better understand the occurrence of CD in WBS. Other studies or longer follow-up might be useful to clarify this issue.

## Background

Williams-Beuren syndrome (WBS) is a well-recognised disorder, with an incidence of approximately 1:10000 live births, and is characterised by typical facial dysmorphisms, heart defects, short stature, and mental retardation [1]. Over 90% of patients present a hemizygous deletion of the elastin gene at 7q11.23 [2,3].

Although coeliac disease (CD) has been reported in patients affected by different chromosomal disorders, including Down syndrome (DS) and Turner syndrome (TS) [4,5], this association has been reported only in sporadic patients with WBS [6-8]. Indeed, we are aware of only two studies in which the prevalence of CD was assessed in a representative series of WBS patients [9,10], and the results were conflicting.

Furthermore, the coexistence and/or prevalence of other autoimmune diseases and organ-specific autoantibodies has not or has rarely been reported in these subjects [11,12].

This study was performed to evaluate longitudinally the prevalence of CD, autoimmune thyroid diseases, and the more frequent organ- and non-organ specific autoantibodies, and to examine HLA typing for the diagnosis of CD in a cohort of patients with WBS.

## Methods

Between June 2003 and May 2011, the prevalence of CD, autoimmune thyroid diseases, and the more frequent organ- and non-organ specific autoantibodies was longitudinally evaluated in 46 (21 from Tuscany, 23 from south Italy-Campania, one from Albania, and one from Chile) consecutive patients with WBS (30 females and 16 males; median age at the onset of the study was 15.4years [range: 4.2–35.3years]). The patients were recruited by the Genetics and Molecular Medicine Unit at Meyer Children’s University Hospital in Florence, Italy, and by the Medical Genetics Unit at San Giuseppe Moscati Hospital, in Avellino, Italy.

The diagnosis of WBS was achieved by the clinical phenotype assessed by two experienced medical geneticists and confirmed by fluorescent *in situ* hybridisation (FISH) results for elastin deletion at 7q11.23. All of the subjects with WBS in this study exhibited typical microdeletions of approximately 1.55 Mb. A minimum of 10 meta-phases was scored for deletion of the 7q11.23 region in each patient. In some cases, array CGH (44 K array platform Agillent oligonucleotides with a resolution of approximately 100 kb) was performed to analyse and precisely map the position of each deletion in this region, and confirmed the minimal region of loss of 1.55 Mb.

Demographic data, family history of autoimmune disorders up to second-degree relatives, drugs administered, height, weight, and BMI were recorded for all patients, and the following antibodies were evaluated: anti-thyroglobulin (TgA), anti-thyroid peroxidase (TPOA), anti-TSH receptor (TSHr), gliadin IgG/A (AGA), anti-endomysium (EmA), and tissue-transglutaminase (tTG) IgA.

Additionally, insulin autoantibodies (IAA), glutamic acid decarboxylase (GAD) and tyrosine phosphatase 2 (IA-2) antibodies, islet cell antibodies (ICA), anti-adrenal cortex antibodies, anti-gastric parietal cell antibodies, antinuclear antibodies (ANA), antismooth muscle antibodies (ASMA), and antiphospholipid antibodies (APA) were also evaluated for 21 patients. All autoantibodies were evaluated annually or at least three times during the study.

The study was approved by the Ethics Committees of Anna Meyer Children’s University Hospital of Florence, and San Giuseppe Moscati Hospital in Avellino. Informed written consent was obtained from the patients and controls and/or their parents.

### Familial autoimmunity

All patients and/or parents were interviewed about their family history, to their second-degree relatives, of autoimmune diseases. As previously described [13], the following autoimmune diseases were considered during the family history: autoimmune thyroid diseases, rheumatoid arthritis and other rheumatologic disorders, CD, type 1 diabetes mellitus, vitiligo, alopecia, multiple sclerosis, and inflammatory bowel disease.

### Thyroid antibodies

Thyroid autoimmunity was evaluated by measuring TPOA and TgA levels using fluorescence enzymatic immunoassays. The normal range was < 35 U/mL for TgA and < 20 U/mL for TPOA.

TSHr autoantibodies were measured with THBIA (DiaSorin spa, Vercelli, Italy) using a two-step radioreceptor assay; TSHr was considered positive at values of > 9 U/L.

### Coeliac disease

All patients underwent an evaluation of serum IgA, IgG/A AGA, EmA, and tTG antibodies. Immunoglobulin (Ig) concentrations were measured by enzyme-linked immunosorbent assay (ELISA) (IgA-human-ELISA quantitation kit). IgA and IgG AGA were measured by ELISA as well. EmA was assayed by a standard immunofluorescence method using cryostat sections of monkey oesophagus. Serum IgA tTG antibodies were assayed with a specific ELISA. Diagnosis of CD was confirmed by performing a small intestine biopsy if the specific autoantibody profile was positive.

### Other antibody assays

ICA was determined by indirect immunofluorescence on cryosections of monkey pancreas using undiluted sera (Inova Diagnostics, Inc., San Diego, CA, USA). GAD, IAA and anti-IA-2 antibodies were measured by radioimmunoassay (RIA) (RSR Ltd, Cardiff, UK).

Anti-adrenal cortex and anti-gastric parietal cell antibodies were detected by indirect immunofluorescence using monkey adrenal glands or gastric mucosa tissues as the substrates (Inova Diagnostics, Inc., San Diego, CA, USA). However, ANA was detected using an immunofluorescent method with HEp-2 cell (Scimedx Corp., Denville, NJ, USA).

ASMA was detected by indirect immunofluorescence using cryostat frozen sections of rat kidney (DiaSorin® Deutschland GmbH - Dietzenbach, Germany). Finally, IgG and IgM APA determination was performed using a standardised ELISA (Diagnostica STAGO, Paris, France).

### HLA typing

Blood (1–9 mL) was collected from all subjects, and DNA for HLA-DQA1 and DQB1 genotyping was extracted. The PCR-amplified exon 2 amplicons were gene-rated for low- to medium-resolution typing in a combined single-stranded conformation polymorphism (SSCP)/heteroduplex assay by a semi-automated electrophoresis and gel-staining method (Protrans HLA Celiac Disease Domino System HLA CD Association kit, Nuclear Laser medicine, Italy). Alleles DQA1*05 and DQB1*02 (encoding the HLA-DQ2 heterodimer) and alleles DQA1*03 and DQB1*0302 (encoding the HLA-DQ8 heterodimer) could be reliably characterised in both the homozygous and heterozygous states.

### Control groups

Data from the 46 WBS patients were compared with the data from 176 healthy age- and sex-matched control subjects (109 females and 67 males; median age of 15.2 years [range: 5.3–36.5 years]) admitted to the hospital for minor surgery (i.e., adenotonsillectomy, phimosis, dermoid cyst, and herniotomy). With regard to the prevalence of HLA typing for the diagnosis of coeliac disease, we compared the WBS patients with 343 healthy adults blood donors (aged between 18–58 years; mean age of 38.8 ± 8.5years).

To determine the prevalence of autoimmune thyroid diseases and CD, the data were also compared to two groups of patients age- and sex-matched that were affected by DS (36 females and 21 males; median age of 15.1years [range: 4.9–23.4years]) and TS (32 females; median age of 16.2years [range: 5.2–40.1years]).

#### Statistical analysis

Statistical analysis was performed using the SPSS statistical package (SPSS Inc., Chicago, IL). Summaries of continuous variables were given as the means ± SDs or median and range, depending on whether the data were normally distributed or not. When appropriate, the χ^2^ test or Fisher’s exact test was used to compare differences between cases and controls. Where appropriate, Bonferroni’s correction for multiple comparisons was applied. Inter-group comparisons for parameters were conducted using analysis of variance (ANOVA) or using repeated-measures analysis of covariance (ANCOVA), as appropriate. Statistical tests were considered significant when p < 0.05.

## Results

The characteristics of controls and WBS, DS, and TS patients are shown in Table 1.

**Table 1:** Demographic data, familial occurrence of autoimmune diseases, organ- and non-organ-specific autoantibodies, and coeliac disease prevalence in patients with Williams-Beuren syndrome, Down syndrome, Turner syndrome, and healthy controls

	**WBS patients**	**Down patients**	**Turner patients**	**Healthy controls**
Subjects (n)	46	57	32	176
Males: females	16:30	21/36	32	67:109
Median age (yr)	15.4	15.1	16.2	15.2
Familial occurrence of autoimmune diseases	13%	-	-	8.6%
Confirmed coeliac disease	2.2%	10.5%*°°	9.4%*^§§^	0.6%
Gliadin IgG autoantibodies	17.4%^^^	19.3%°°°	15.6%^§§§^	5.1%
Gliadin IgA autoantibodies	2.2%	12.3%**°°	12.5%**^§§^	1.7%
Endomysium autoantibodies	2.2%	10.5%*°°	9.4%*^§§^	0.6%
Tissue-transglutaminase autoantibodies	2.2%	12.3%**°°	9.4%*^§§^	0.6%
Thyroid autoantibodies	0.0%	10.5%***°°	9.4%**^§§^	1.1%
Thyroglobulin autoantibodies	0.0%	7.0%*°	6.2%*^§^	1.1%
Thyroid peroxidase autoantibodies	0.0%	10.5%***°°	9.4%**^§§^	0.6%
TSH receptor autoantibodies	0.0%	1.7%	0.0%	0.0%
Organ- and non-organ specific autoantibodies	0.0%	7.0%*	9.3%*^§^	2.3%
Insulin autoantibodies	0.0%	0.0%	0.0%	0.0%
Glutamic acid decarboxylase autoantibodies	0.0%	1.75%	0.0%	0.0%
Tyrosine phosphatase 2 autoantibodies	0.0%	0.0%	0.0%	0,6%
Islet cell autoantibodies	0.0%	0.0%	0.0%	0.0%
Adrenal cortex autoantibodies	0.0%	0.0%	0.0%	0.0%
Gastric parietal cell autoantibodies	0.0%	1.75%	3.1%	0.0%
Antinuclear antibodies	0.0%	1.75%	6.2%^§§§^	1.1%
Smooth muscle autoantibodies	0.0%	0.0%	0.0%	0.6%
Phospholipid autoantibodies	0.0%	1.75%	0.0%	0.0%

WBS, Williams-Beuren syndrome; *WBS *vs.* Down syndrome or Turner syndrome: *p < 0.05; **p < 0.005; ***p < 0.0005; ^WBS *vs.* controls: ^p < 0.05; ^^p < 0.005; ^^^p < 0.0005; °Down syndrome *vs.* controls: °p < 0.05; °°p < 0.005; °°°p < 0.0005; ^§^Turner syndrome *vs.* controls: ^§^p < 0.05; ^§§^p < 0.005; ^§§§^p < 0.001.

At the time of the end of the study, the mean follow-up of the WBS patients was 7.4 ± 0.8 years (range 5.6 - 8.5 years). Autoantibodies were evaluated 6.2 ± 1.8 times during the study, without differences among the determination of CD autoantibodies (7.4 ± 1.0 times), thyroid autoantibodies (6.1 ± 1.8 times), and other autoantibodies (6.6 ± 1.5 times).

Six (13%) WBS patients had a positive family history for autoimmune diseases, three with autoimmune thyroid disease, two with rheumatoid arthritis, and one with CD, though with no significant difference with respect to the controls (8.6%; P = NS). No patients showed an IgA deficiency.

Regarding the prevalence of CD, only one WBS patient (a male, 2.2%) was found to be positive for coeliac antibodies; the diagnosis of CD was confirmed histologically. However, this patient had a strong positivity for autoimmune diseases in first relatives (autoimmune thyroid disease in the mother and sister and CD in one maternal cousin). Although the data for CD in WBS were not significantly different from the healthy controls (0.6%; P = NS), the data were significantly different from DS (10.5%; P < 0.005) and TS (9.4%; P < 0.005).

In contrast, no patient with WBS tested positive for TPOA, TgA, or TSHr, which was significantly different from the DS (10.5%; P < 0.005) and TS (9.4%; P < 0.005) patients, but not from the healthy controls (1.1%; P = NS).

Finally, the overall frequency of other organ- and non-organ specific autoantibodies did not significantly differ among WBS patients and the controls (0.0% *vs.* 2.3%), but it was significantly different from DS and TS (7.0% and 9.3%, respectively, P < 0.05).

Coeliac-specific HLA-DQ heterodimers were present in 20 patients (43%), though the frequency of each haplotype were not significantly higher than those of the controls (43%; P = NS). In particular, the DQ2 and DQ8 haplotypes were positive in sixteen (34% *vs.* 33%; P = NS) and four patients (9% *vs.* 10%; P = NS), respectively.

Twenty-six patients (57%) tested negative for coeliac-specific HLA-DQ heterodimers, but showed the DQA1*0505 allele. This frequency was statistically significant with respect to the controls (39%; P < 0.05).

## Discussion

Our data show that patients with WBS do not develop CD any more significantly than the general population but less frequently than patients with DS and TS. In addition, the WBS patients did not show a higher prevalence of anti-thyroid autoantibodies and other autoantibodies than the healthy controls, and their prevalence was significantly reduced in comparison to those with TS and DS.

Therefore, our data appear to negate the utility of assessing the main autoimmune diseases and organ- and non-organ specific autoantibodies in WBS, as opposed to other genetic disorders that are associated with a higher prevalence of autoimmune diseases [14-16].

In a previous cross-sectional study [12] of patients with WBS, and in agreement with other reports [17-19], we found that these patients did not present positivity for anti-thyroid autoantibodies. Nonetheless, other authors have described rare cases of autoimmune thyroid disease [11].

The data from the present longitudinal study confirm our previous results [12]. However, our study did not find a significant prevalence of CD. For comparison, Giannotti *et al.*[10] reported a prevalence of CD in 9.5% of WBS patients, whereas Santer *et al.*[9] reported only one (1.4%) WBS patient affected by CD in a series of 71 children undergoing specific screening. Our results, in agreement with those of Santer *et al.*[9], may suggest that the association of WS and CD is rare.

Nevertheless, the relatively small sample size may be a limit of our study. However, compared to the two aforementioned cross-sectional studies, our study represents the first long longitudinal evaluation of autoimmunity in the WBS patients, permitting to better capture the history of autoimmune disorders in these syndrome.

Despite the different sample size (71 patients in the Santer *et al.* study, 63 patients in the Giannotti *et al.* study, and 46 patients in the present study), the similar median age and age range are common characteristics of these three studies. Moreover, the two previous studies not used anti-tissue transglutaminase antibodies, a useful screening marker for CD. Also, both previous studies did not present a genetic study of HLA predisposition of the patients.

We do not know because the study of Giannotti *et al.* showed a very high frequency of CD with respect to our data and the data of Santer *et al.*[9]. We may hypothesize that other causes of small bowel biopsy lesions may be associated with diarrhea or malabsorption, even if only CD responds to a gluten-free diet, such as infectious disorders, deficiency of zinc, vitamin B12, and acid folic, drug-induced small bowel injury [20].

Furthermore, WBS patients appear to not show a more frequent prevalence of other autoimmune disorders in addition to CD. This lack of correlation is clearly different from other genetic syndromes related to autoimmune disorders, such as TS and DS.

TS, one of the most common sex chromosome abnormalities [21] that is caused by the presence of only one normally functioning X-chromosome, has a more frequent prevalence (4.2–6.4%) of different autoimmune diseases, such as autoimmune thyroid diseases and anti-thyroid antibodies with CD [16,22,23]. Other autoimmune diseases described include inflammatory bowel disease, insulin-dependent diabetes mellitus (IDDM), Addison's disease, rheumatoid arthritis, myasthenia gravis, vitiligo, alopecia, pernicious anaemia, and hypoparathyroidism [24].

There is also strong evidence for an association between DS and CD, with prevalence between 5% and 12% [15]. Autoimmune thyroid diseases are the most frequent autoimmune disorder coexisting with this syndrome, and most of the affected patients (varying from 3 to 28%) display detectable circulating thyroid-specific autoantibodies [25]. An increased prevalence of type 1 diabetes mellitus over the general population has also been described in these patients [25].

However, despite the data reported in a previous study [10] regarding the higher prevalence of CD in WBS, there are no available studies or protocols on the follow-up of autoimmune disease in these patients, which is in contrast to the situation for patients with TS and DS.

Among autoimmune disorders, an increased prevalence of CD and autoimmune thyroid diseases has been found in patients with autoimmune thyroid disease, type 1 diabetes mellitus, autoimmune liver diseases, and inflammatory bowel disease [26]. Thus, a similar genetic predisposition is common to many autoimmune diseases, and the genes in the HLA complex are among the strongest predisposing genetic factors [27].

Recent population screening studies have shown that the prevalence of CD in Western countries approaches 1%. There is considerable genetic influence in CD; 90–100% of affected individuals possess either the class II HLA molecule DQ2 (DQA1*05/DQB1*02) and/or DQ8 (DQA1*0301/DQB1*0302) compared to 30–40% of the general population [28,29].

Furthermore, whereas some authors have not provided evidence of an association with CD and the ELN17 marker [30], our data suggest that WBS patients exhibit the DQA1*0505 allele more frequently that controls (56% *vs.* 39%; P < 0.05). The DQA1*0505 allele is similar to the DQA1*0501 allele of the DQ2.5 haplotype [31]. However, some genetic studies have revealed that the DQA1*0505:X/Y:DQB1*0202 gene products may explain disease not linked to the haplotype that produces DQ8 and DQ2.5 [32].

## Conclusion

In conclusion, no evidence of a significantly higher prevalence of autoimmune diseases or positivity of the main organ and non-organ specific autoantibodies was found in subjects with WBS. This result appears to be different with respect to other genetic syndromes (e.g., TS or DS), in which there is a major frequency of certain autoimmune disorders. This should prompt us to better understand the occurrence of CD in the WBS. Accordingly, we suggest that routine examination of autoimmune diseases should not be performed in the follow-up of asymptomatic WBS patients. Other studies or longer follow-up might be useful to clarify this issue.

## Competing interests

The Authors declare that they have no competing interests.

## Authors’ contributions

SS carried out the endocrinological evaluation, conceived the study and participated in its design. EL, carried out the clinical genetic diagnosis. GP carried out the immunological evaluation. SR carried out the immunological evaluation. CA carried out the immunological evaluation. MGA carried out the clinical genetic diagnosis. SG carried out the clinical genetic diagnosis. SR carried out the clinical genetic diagnosis. SS carried out the endocrinological evaluation. FC participated in the endocrinological evaluation. MM participated in the endocrinological evaluation and participated in its coordination. All authors read and approved the final manuscript.

## Pre-publication history

The pre-publication history for this paper can be accessed here:

http://www.biomedcentral.com/1471-2350/15/61/prepub
